# Spark Plasma Sintering of LiFePO_4_: AC Field Suppressing Lithium Migration

**DOI:** 10.3390/ma14112826

**Published:** 2021-05-25

**Authors:** Nan Luo, Yong Lin, Jian Guo, Emanuele Quattrocchi, Huaijiu Deng, Jian Dong, Francesco Ciucci, Filippo Boi, Chunfeng Hu, Salvatore Grasso

**Affiliations:** 1 Key Laboratory of Advanced technologies of Materials, Ministry of Education, School of Materials Science and Engineering, Southwest Jiaotong University, Chengdu 610031, China; luonan95@outlook.com (N.L.); linyong1965726725@outlook.com (Y.L.); denghuaijiu9@outlook.com (H.D.); dongjian1996318@outlook.com (J.D.); chfhu@live.cn (C.H.); 2 College of Physics, Sichuan University, Chengdu 610064, China; jianguo@scu.edu.cn (J.G.); f.boi@scu.edu.cn (F.B.); 3 Department of Mechanical and Aerospace Engineering, The Hong Kong University of Science and Technology, Hong Kong, China; equattrocchi@connect.ust.hk (E.Q.); francesco.ciucci@ust.hk (F.C.); 4 Department of Chemical and Biological Engineering, The Hong Kong University of Science and Technology, Hong Kong, China

**Keywords:** spark plasma sintering, LiFePO_4_, DC and AC, lithium-ion migration

## Abstract

Our work proposes a comparison between Spark Plasma Sintering of LiFePO_4_ carried out using an Alternating Current (AC) and Direct Current (DC). It quantifies the Li-ion migration using DC, and it validates such hypothesis using impedance spectroscopy, X-ray photoelectron spectroscopy and inductively coupled plasma optical emission spectroscopy. The use of an AC field seems effective to inhibit undesired Li-ion migration and achieve high ionic conductivity as high as 4.5 × 10^−3^ S/cm, which exceeds by one order of magnitude samples processed under a DC field. These results anticipate the possibility of fabricating a high-performance all-solid-state Li-ion battery by preventing undesired Li loss during SPS processing.

## 1. Introduction

Li-ion batteres LIBs could power an endless number of devices, including electric vehicles (EVs) [1,2,3]. At present, LIBs applications are limited by their safety, cost, stability/reliability and volumetric energy density [4]. Lithium iron phosphate (LiFePO_4_) is one of the few well-established materials for LIBs cathode that does not contain rare earth. It has a moderate working voltage (3.4 V vs. Li/Li^+^) and high specific capacity (170 mAh/g) [5,6,7]. Moreover, the cell balancing is relativity simple due to its flat potential curve. Its low density contributes to a high specific capacity in the order of 170 mAh/g matching Lithium Cobalt Oxide (LiCoO_2_). Compared with LiCoO_2_, LiNiO_2_, LiMn_2_O_4_ and its related cathode materials, LiFePO_4_ has outstanding advantages in terms of cost, high-temperature performance and safety. Because of this, it is expected to become one of the preferred cathode materials for medium-to-large capacity and medium-to-high power lithium-ion batteries (5 Ah–1000 Ah).

Currently, LFP is employed in the presence of liquid electrolytes, such as lithium fluoride, dimethyl carbonate, diethyl carbonate and lithium hexafluorophosphate [8,9]. However, these electrolytes are in most cases flammable and/or toxic. Because of this, the current research is focused on the solid-state battery, Wang et al. [10] reported on a new type of solid-like electrolyte (SLE) integrated into the rechargeable Li|LiFePO_4_ solid-state battery (SSB), with a loading of 25 mg/cm^2^ and excellent performance in the temperature range from −20 to 150 °C. Chen et al. [11] produced a Li/LLZO/LiFePO_4_ full battery with a discharge capacity of 120 mAh/g for LiFePO_4_ and 2200 mAh/g with Si anodes operating at room temperature. The capacity retention rate reached 72% after a cycle of 100 times. Currently, only a few emerging processing techniques, including SPS [12], cold sintering [4,13] and flash sintering, seem suitable to produce dense LFP. Processing of SSB remains rather challenging because of thermally activated lithium volatilization and the undesired inter-diffusion between the layers (i.e., anode, electrolyte and cathode) [14,15]. In this context, SPS is a well-established field-assisted sintering technique, which emerged at the beginning of the 1990s. It employs a uniaxial pressure and a pulsed DC discharge allowing heating rates in excess of 100 °C/min [16,17]. The previous studies were carried using a DC current. The development of asymmetric microstructures [18,19], usually developed under a DC field, is justified the ionic migration by the chemical interactions with defects in the cathode region for ionic conductors and by the asymmetric temperature distribution.

When comparing SPS to flash sintering, these effects are even more pronounced in the latter case because of the increased voltage drop across the specimen. However, even under SPS conditions, the application of DC field below 10 V seems to induce some polarity effects as described in Ref. [20]. Pronounced temperature gradients are associated with the processing of thermoelectric materials [21,22]. Very recently, Tarini et al. [23] also reported SPS reducing conditions (including the direct contact between the sample and graphite) results in large internal stresses causing the formation of macroscopic cracks.

To the best of our knowledge, these polarities induced effects have not been extensively investigated in the case of Li-ion conductors. These effects are expected to be more severe when the thickness of the sample is reduced down to 50–100 µm interlayers (i.e., desired target for SSB) as the migration path is involved. SPS consolidation has been historically carried using DC or pulsed DC because of the simplicity and reduced equipment cost (inverters, rectifiers and controllers). In this study, we carried a comparative analysis on SPS LFP samples produced using DC and AC waveforms. The electrochemical impedance spectroscopy (EIS) and X-ray photoelectron spectroscopy (XPS) analysis confirmed the detectable of lithium-ion migration under the DC field.

## 2. Materials and Methods

SPS processes using DC and AC fields. Commercially available LFP powder (LFP-NCO, Aleees, Taiwan) with the grain size (2–4 μm) contained about 1.9 wt% carbon. Powders were poured inside graphite die under a pressure of 30 MPa and heated from room temperature to 700 °C using a rate of 50 °C/min with 10 mins dwelling time with DC and AC currents, then release pressure and cool naturally to room temperature. In order to identify specific effects induced by the DC and AC, the powders were processed using identical experimental conditions. The temperature was measured using a K-type thermocouple, and the inherent temperature profile is shown in Figure S1. The powder was sintered using the spark plasma sintering (SPS) technique (ZT-50-24Y, Chenhua Corp., Shanghai, China) with DC current. A custom-built SPS unit (CXWK022020, Chenxin Corp., China) was used to compare SPS LFP samples produced using DC and AC current. 

The density of the sintered samples was determined according to the Archimedes method. The XPS studies were performed using an AXIS Supra (Kratos) Spectrometer using monochromatized Al-K_α_ radiation. SEM (FEI Inspect F50 (FSEM)) was used to observe microstructures of the fractured cross-section of LFP pellets at different sample locations to account for the polarity effects. Inductively coupled plasma optical emission spectroscopy (ICP-AES) (Agilent 720) was performed on the LFP sintered samples to determine the lithium content. X-ray diffraction (XRD) was conducted using an Empyrean diffractometer (Co-Kα radiation) within the 2θ range of 5–85°, as shown in Figure S2. Raman scattering experiments were carried out on a custom-built confocal Raman spectrometry system in the backscattering geometry based on a triple-grating monochromator (Andor Shamrock SR-303i-B) with an attached EMCCD (Andor Newton DU970P-UVB). A spectral resolution of ±1 cm^−1^ was reached, and the spatial resolution was ±1 μm. The excitation line at 532 nm was produced by a laser source (RGB laser system) focused on the sample using a Mitutoyo™ 50× working distance objective (0.28 N.A.). The laser power at the sample was 50 mW. The Raman results are reported in Figure S3.

The pellets were cut into cuboids of 6 × 6 × 1 mm^3^. The DC SPS samples were sectioned from the anode and cathode SPS regions, while the AC SPS was collected from the analogous portions of the sample. The electronic/ionic conductivities of the sintered samples were measured at 30 °C using an Electrochemical Workstation (CS2350H, Correst Corp., China) in a controlled temperature chamber with a frequency ranging from 10^6^ Hz to 1 Hz and sine wave signal with an amplitude of 20 mV. Impedance analysis results are detailed in the Supplementary Materials. 

## 3. Results

**Figure 1:**
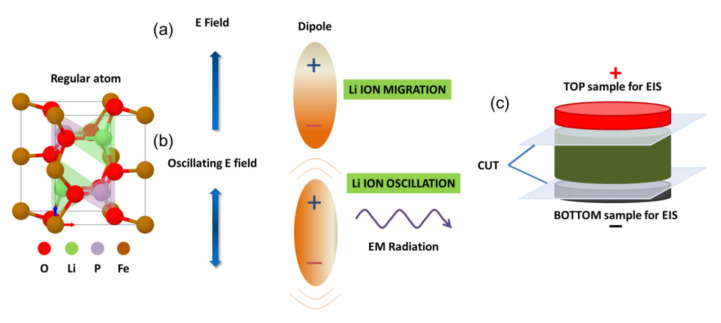
A schematic diagram of the SPS working setup using a comparison of Li-ion migration effects under (**a**) DC and (**b**) AC fields, (**c**) a cross-section of the sample for SEM, impedance spectroscopy, XPS and XRD.

A schematic diagram of the SPS working setup using DC and AC fields is shown in Figure 1. The current flows from the positive electrode to the negative electrode, and at the same time, the migration of positively charged lithium ions might also be expected. On the other side, the AC field results in Li-ion oscillation rather than net migration. Ion migration is expected to become severe during the sintering as the ionic conductivity has an exponential dependence with respect to the temperature. The typical voltage drop across the sintering sample is below 1 V [24], which might be sufficient to generate an undesired ionic migration. Most of the SPS machine would typically employ graphite tooling, which is a non-blocking electrode capable of hosting the mobile Li-ions. In order to suppress the loss of Li inside the graphite punches, another viable option could be to electrically insulate the sample and thus losing any possible field-induced effect. To the best of our knowledge, these aspects were not investigated in earlier works. 

**Figure 2:**
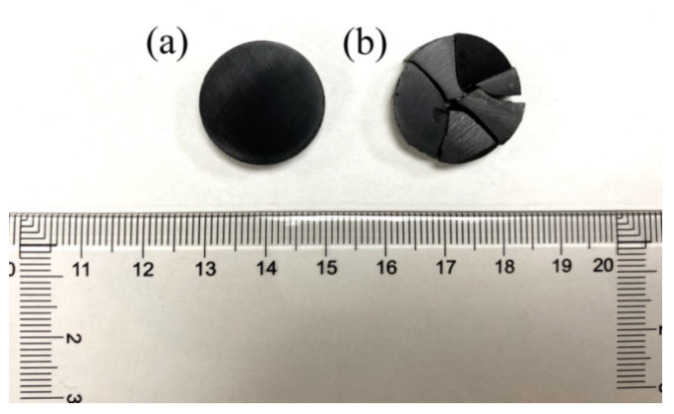
An image of LiFePO_4_ samples processed using SPS with (**a**) AC and (**b**) DC (Ø 20 mm). The DC SPS samples consistently cracked because of the volume phase change during SPS.

It can be seen in Figure 2 that the sample cracked when processed under DC. On the contrary, the sample maintained its integrity when using AC. These results were repeated several times, giving a reproducible outcome. In both AC and DC, the samples were processed under identical heating and cooling profiles (see Figure S1) and identical geometry of the punches/die assembly; therefore, the cracking should not be ascribed to a thermal shock effect. Under the DC field, the migration of lithium ions may cause the sintering bulk to crack due to the phase volume change occurring during the ionic migration. During the intercalation process, the phase transition from lithium-poor (FePO_4_) to lithium-rich (LiFePO_4_) causes a volumetric change (i.e., a-direction (*ε_a_* = 5.03%), while shrinkage occurs in the c-direction (*ε_c_* = −1.9%) and *ε_b_* = 4.5% [25]). The resulting stress may explain the formation of cracks [26] seen for samples processed under DC. 

SPS employed graphite punches that are not Li-blocking. As a result, under a DC field, a Li migration from the sample to the punch should be expected [27], and the DC field also caused undesired cracking of the samples [28]. Inversely, SPS in AC 50 Hz resulted in uncracked samples because of the blocking effect of the AC field [29]. 

**Figure 3:**
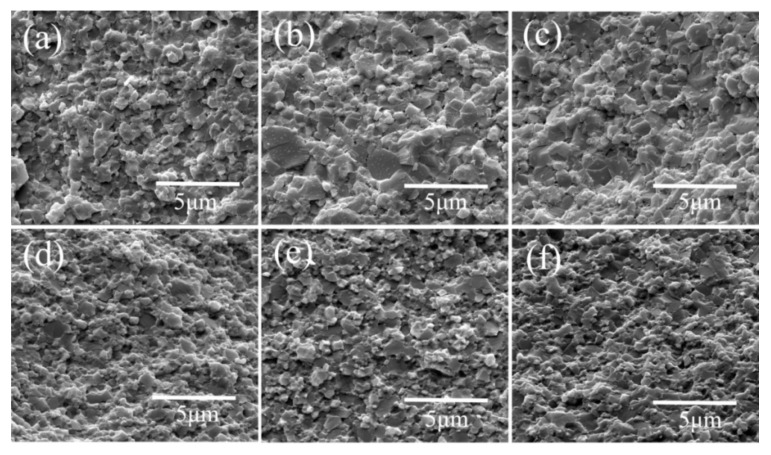
Comparative analysis showing the SEM images of LiFePO_4_ samples: (**a**) LFP-SPS-AC-Top, (**b**) LFP-SPS-AC-Middle, (**c**) LFP-SPS-AC-Bottom, (**d**) LFP-SPS-DC-Top, (**e**) LFP-SPS-DC-Middle, (**f**) LFP-SPS-DC-Bottom.

Apart from this practical aspect related to the integrity of the sample, our study aimed to clarify the possible ion loss and other effects induced by a DC and AC field. The cross-sectional SEM images of LFP cathodes prepared with SPS using AC and DC are shown in Figure 3a–f. Based on SEM observations of the fractured surfaces, it was hypothesized that the small grain size could be maintained. All sintered LFP samples with SPS had a high density of 94.3% (LFP-AC) and 95.5% (LFP-DC). Regardless of the AC or DC processing, there was almost no difference in the microstructure of the top and bottom morphological images of the samples shown in Figure 3. The microstructures of LFP-AC and SPS-AC samples were characterized by a bimodal grain size distribution with a slightly increased grain size [30]. Further work is still needed to clarify the grain growth behavior when comparing AC and DC fields. In addition, regardless of the application of the of an AC or DC field, LFP grains fractured in intergranular and transgranular mode. The latter indicated grain boundaries had a good mechanical strength [16]. 

**Figure 4:**
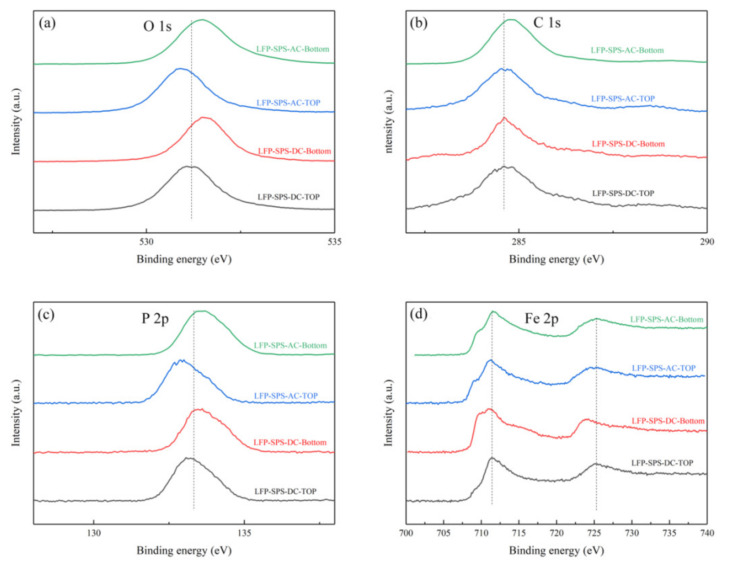
The high-resolution XPS spectra of (**a**) C 1s, (**b**) O 1s, (**c**) P 2p, and (**d**) Fe 2p in different samples.

**Figure 5:**
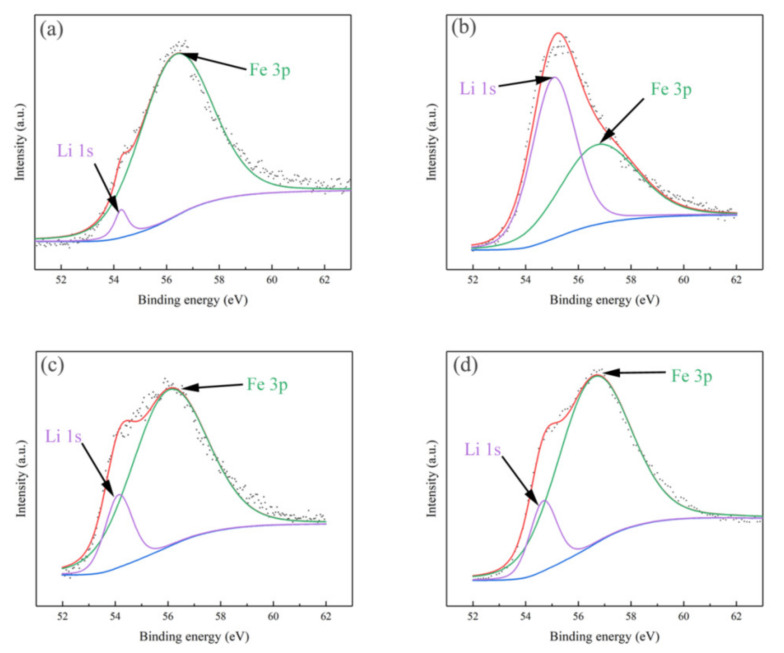
The high-resolution XPS spectra of Fe 3p–Li 1s in different samples: (**a**) LFP-SPS-DC-Top, (**b**) LFP-SPS-DC-Bottom, (**c**) LFP-SPS-AC-Top and (**d**) LFP-SPS-AC-Bottom.

The XPS spectra of LiFePO_4_ samples in the binding energy range of O 1s, C 1s, Fe 2p and P 2p are shown in Figure 4. All of the O 1s spectra displayed a narrow peak at 531.4 eV, which was attributed to oxygen atoms of the (PO_4_)^3-^ groups [31]. The C 1s peak of all samples was detected at 284.6 eV, which was the carbon [32]. The binding energy peak of P 2p was located at 133.9eV characterizes the presence of this unit with P^5+^ [33]. In the XPS spectrum of Fe 2p, the binding energies of Fe^2+^ were 709.8 eV and 724 eV, respectively; the binding energies Fe^3+^ ware 711.9 eV and 726 eV [31,34]. Figure 4d shows that Fe was almost totally at the +III oxidation state at the LFP-SPS-DC-Top since the lithium ions migrated to the negative electrode [31]. However, the relative sensitivity factor of Li 1s is about 30 times smaller than Fe 3p. Therefore, as shown in Figure 5, for each sample, the spectra of Fe 3p and Li 1s were respectively fitted. There are two peaks around ~55 eV, corresponding to Fe ^2+^ and Li ^+^ in LiFePO_4_ respectively [35]. In the DC sample, the peak area ratio of Li 1s to Fe 3p in the positive electrode part was significantly lower than that in the negative electrode part. On the contrary, when AC was used, the area ratio of the top to the bottom was similar. Those results further confirmed the lithium ion migration phenomenon in the LFP cathode during the DC SPS sintering, while such effect is not seen in the case of an AC waveform. 

**Figure 6:**
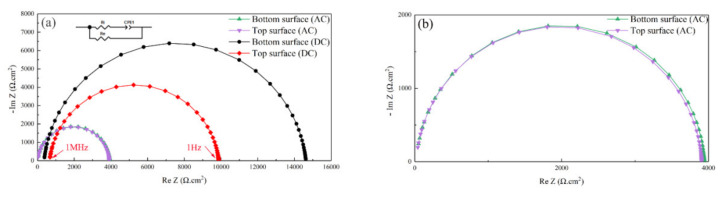
(**a**) Nyquist Plot recorded at 30 °C for LiFePO_4_ samples consolidated using DC and AC and (**b**) its enlarged image.

Impedance spectroscopy was employed to confirm the Li migration depending on the AC or DC SPS field. Nyquist plots of LiFePO_4_ samples are shown in Figure 6. The set of plots reveals a slightly concave semicircular arc and fitted with the equivalent circuit [36] shown inset. The *R_i_* component represents the ionic ohmic resistance, and the *R_e_* component represents the electronic ohmic resistance. According to the equivalent circuit inserted in Figure 6, the parallel *RiRe*/*Ri + Re* is the intersection of the high frequency 1 MHz line and the real axis, and the electronic resistance *Re* is the intersection of the real axis and1 Hz. The electronic (*σ_e_*) and ionic (*σ_i_*) conductivity of the LiFePO_4_ samples is calculated after fitting the curves in Figure 6 (listed in Table 1). The ionic conductivity of the DC sample at the top (1.41 × 10^−4^ S/cm) was lower than that at the bottom (2.69 × 10^−4^ S/cm). Instead, the electronic conductivity of the top surface (1.01 × 10^−5^ S/cm) was significantly higher than the bottom (6.84 × 10^−6^ S/cm). This is probably due to the different Li content of the top and bottom samples, which could change *σ_e_* [37]. The inductively coupled plasma-optical emission (ICP) results further confirmed that lithium-ion migration occurred during the SPS technique using DC. Oppositely, the respective ionic conductivity (4.42 × 10^−3^ S/cm and 4.71 × 10^−3^ S/cm) and electronic conductivity (2.54 × 10^−5^ S/cm and 2.56 × 10^−5^ S/cm) of LFP-AC are similar in both portions of the specimen as no asymmetric electric field occurred in AC.

In addition, AC SPS sample with low Li vacancy concentration due to the fast sintering, resulting in high conductivity. The complete high ionic and electronic conductivity LFP electrode prepared using AC provides a reliable sintering technology for the co-sintering of solid-state batteries with excellent electrochemistry performance. For comparison, Elango et al. have reported the use of SPS to sinter LFP cathode materials using a salt templating method [38,40]. The samples in Ref. [38] and other references calculated the electronic and ionic conductivity using an analogous approach proposed in the present work. Comparing the results listed in Table 1, there is little difference in electronic conductivity. Obviously, the ionic conductivity of the AC sample was even two orders of magnitude higher than that reported by Refs. [38,39]. Such difference might be attributed to the low-porosity SPS and the suppressed ionic migration. The results are encouraging to support further investigations on rate capability, cycle stability and low-temperature capability of the AC SPSed LFP cathode materials [39].

## 4. Conclusions

We propose a comparison of spark plasma sintering of LiFePO_4_ using AC and DC, accounting for possible electrochemical effects and lithium-ion migration/non-blocking effects. SEM confirmed that crystalline and well-distributed LiFePO_4_ particles were well sintered by SPS both using AC and DC. During DC SPS sintering, lithium-ion migration was observed, leading to the catastrophic fracture of the LFP specimen. On the other side, preferential lithium-ion migration was not observed during SPS using an AC field, and the sample integrity was retained. The proposed AC SPS approach resulted in ionic conductivity two orders of magnitude higher than one reported in the literature, paving the way for polarity-controlled field-assisted sintering. The comparison between SPS using DC and AC suggests the electric field impacts the ionic conductivity of the processed materials. 

## Figures and Tables

**Table 1:** The electronic (*σ_e_*) and ionic (*σ_i_*) conductivity of the LiFePO_4_ samples and ICP-AES Li content of LFP materials. Literature references’ values, processing route and comparative values are also reported.

Samples		*R_e_* (Ω.cm^2^)	*σ_e_*(S/cm)	*R_i_* (Ω.cm^2^)	*σ_i_*(S/cm)	ICP Li (wt%)
**DC**	Top surface (+)	9879	1.01 × 10^−5^	708	1.41 × 10^−4^	3.85
	Bottom surface (−)	14626	6.84 × 10^−6^	372.2	2.69× 10^−4^	3.89
**AC**	Top surface	3941	2.54 × 10^−5^	22.61	4.42 × 10^−3^	3.92
	Bottom surface	3908	2.56 × 10^−5^	21.19	4.71 × 10^−3^	3.94
**Literature**	DC SPS		(1~4) × 10^−5^ [38]		(3.4~14) × 10^−5^ [38]	
	Cold dry pressing		(3~10) × 10^−5^ [39]		(7~12) × 10^−6^ [39]	

## Data Availability

The data presented in this study are available on request from the corresponding author.

## Supplementary Materials

The following are available online at https://www.mdpi.com/article/10.3390/ma14112826/s1, Figure S1: Pressure and temperature vs. time curve (30 MPa applied pressure, heating rate of 50 °C/min up to 700 °C with 10 min dwelling), Figure S2: The XRD patterns of LiFePO_4_ samples under SPS using AC and DC, Figure S3: Raman spectra of LiFePO_4_ samples under SPS using AC and DC (The main peaks are at 950, 990, 1100 cm^−1^), Figure S4: Nyquist fitting diagrams of the EIS data of LFP-AC-TOP—(A) Wang model, (B) ZARC model method, LFP-AC-Bottom—(C) Wang model, (D) ZARC model method, LFP-DC-TOP—(E) Wang model, (F) ZARC model method, and LFP-DC-Bottom—(G) Wang model, (H) ZARC model method, Table S1: The formula from the methods.
