# Supplementary material for: Spark Plasma Sintering of LiFePO4: AC Field Suppressing Lithium Migration
Source: Materials (Basel). 2021 May 25;14(11):2826. doi: 10.3390/ma14112826 (PMC8198947; doi:10.3390/ma14112826)
Supplement: Supplementary file 1 [file materials-14-02826-s001.zip › materials-1211510-supplementary.pdf]

materials

MDPI

Supplementary section

Spark Plasma Sintering of $LiFePO_4$: AC Field Suppressing Lithium Migration

Nan Luo [1], Yong Lin [1], Jian Guo [2], Emanuele Quattrocchi [3], Huaijiu Deng [1], Jian Dong [1], Francesco Ciucci [3,4], Filippo Boi [2], Chunfeng Hu[1] and Salvatore Grasso [1,*]

1 Key Laboratory of Advanced Technologies of Materials, Ministry of Education, School of Materials Science and Engineering, Southwest Jiaotong University, Chengdu 610031, China; luonan95@outlook.com (N.L.); linyong1965726725@outlook.com (Y.L.); denghuaijiu9@outlook.com (H.D.); dongjian1996318@outlook.com (J.D.); chfhu@live.cn (C.H.), s.grasso@swjtu.edu.cn (S.G.)
2 College of Physics, Sichuan University, Chengdu 610064, China; jianguo@scu.edu.cn (J.G.); f.boi@scu.edu.cn (F.B.)
3 Department of Mechanical and Aerospace Engineering, The Hong Kong University of Science and Technology, Hong Kong, China; equattrocchi@connect.ust.hk (E.Q.); francesco.ciucci@ust.hk (F.C.)
4 Department of Chemical and Biological Engineering, The Hong Kong University of Science and Technology, Hong Kong, China
* Correspondence: s.grasso@swjtu.edu.cn; Tel.: 18328676558

Citation: Luo, N.; Lin, Y.; Guo, J.; Quattrocchi, E.; Deng, H.; Dong, J.; Ciucci, F.; Boi, F.; Hu, C.; Grasso, S. Spark Plasma Sintering of $LiFePO_4$: AC Field Suppressing Lithium Migration. *Materials* **2021**, *14*, 2826. https://doi.org/10.3390/ma14112826

Academic Editor: Evgeny Levashov

Received: 21 April 2021
Accepted: 21 May 2021
Published: 25 May 2021

Publisher's Note: MDPI stays neutral with regard to jurisdictional claims in published maps and institutional affiliations.

Copyright: © 2021 by the authors. Licensee MDPI, Basel, Switzerland. This article is an open access article distributed under the terms and conditions of the Creative Commons Attribution (CC BY) license (http://creativecommons.org/licenses/by/4.0/).

As receive $LiFePO_4$ were processed using SPS under a DC or AC field. The relative temperature profiles are shown in Figure S1.

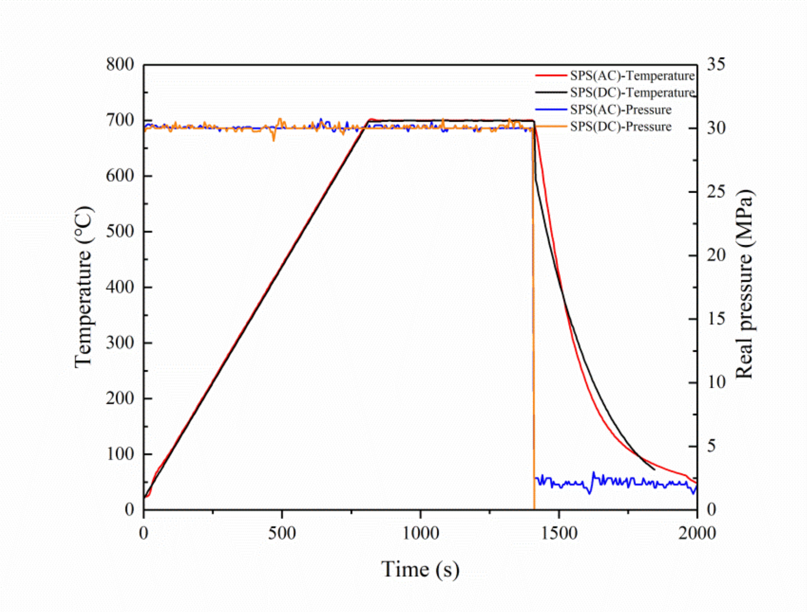

Figure S1. Pressure and temperature vs. time curve (30MPa applied pressure, heating rate of 50 °C/min up to 700 °C with 10 mins dwelling).

X-ray diffraction (XRD) was conducted using an Empyrean diffractometer (Co-K⊚ radiation) within the 2θ range 5°~85°. XRD patterns of LFP powders and LFP cathode sintered by SPS using DC and AC are depicted in Figure S2 (a, b). All observed peaks in LFP samples matched well with the PDF#83-2092 and no impurity peaks were detected [1,2].

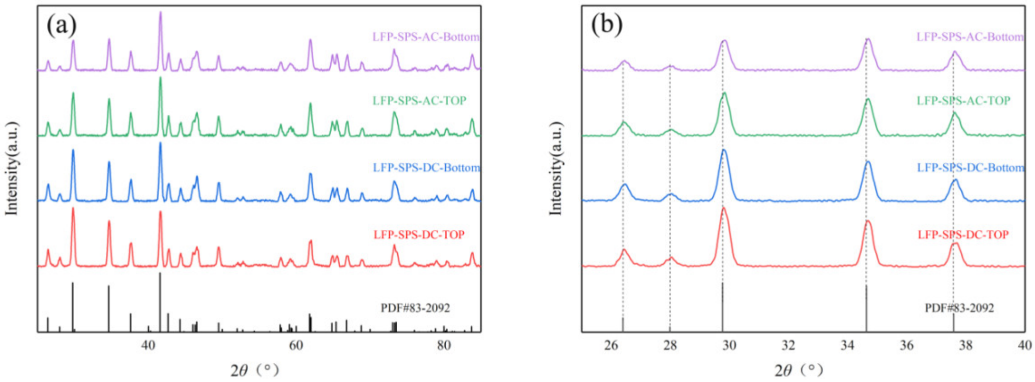

Figure S2. The XRD patterns of $LiFePO_4$ samples under SPS using AC and DC.

Raman scattering experiments were carried out on a custom-built confocal Raman spectrometry system in the backscattering geometry based on triple grating monochromator (Andor Shamrock SR-303i-B) with an attached EMCCD (Andor Newton DU970P-UVB). A spectral resolution of ±1 cm^{-1} was reached, and the spatial resolution was ±1 μm. The excitation line at 532 nm was produced by laser source (RGB laser system) focused on the sample using a Mitutoyo™ 50×, working distance objective (0.28 N.A.). The laser power at the sample was 50 mW.

The Raman spectra reported in Figure S3 with peaks observed in the region of 900–1100 cm^{-1}. It can be seen that the highest intensity peak at 950 cm^{-1} corresponds to the P–O stretching vibration band of ν1. In addition, the two low-strength bands between 990 and 1100 cm^{-1} are due to the asymmetric tensile band of PO_4^{3-} anion (ν_3) [3]. The peak positions in this range reported in Figures S3, the same as those reported in the Ref. [4]. No significant degradation of LFP occurred when using SPS in both DC and AC.

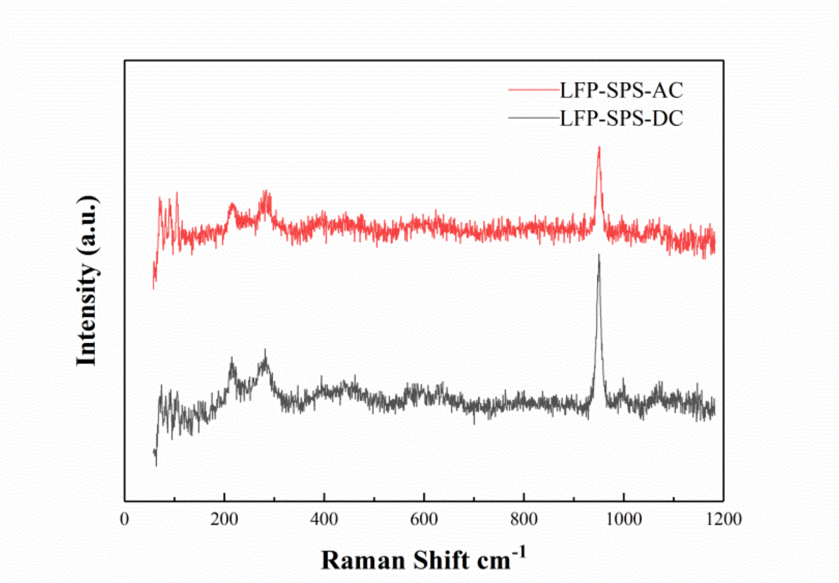

Figure S3. Raman spectra of $LiFePO_4$ samples under SPS using AC and DC (The main peaks are at 950, 990, 1100 cm^{-1}).

We obtained further insights regarding the conductivities of the LFP from the EIS data by applying the Wang [5] model, MATLAB Wang simplified model (ZARC), and distribution of relaxation of times (DRT) analysis. The conductivities from the methods were computed using formula from Table S1. The Nyquist fitting diagrams of the EIS data of LFP as shown in Figure S4. According to the electronic (σ_e) and ionic (σ_i) conductivity of the $LiFePO_4$ samples is calculated after fitting the curves (listed in Table S2), the 2

models (Wang and Simplified Wang) are coherent. However, the DRT estimated moves from the theoretical for the following reason: The loss used in the Machine Learning code is based on the exp EIS data rather than exact Z (computed using the ECM model).

Table S1. The formula from the methods.

Models	$Z(f,\psi)$	$\gamma(f,\psi)$
**ZARC** $(R_i = 0)$ $(\tau_0 = CR_e^{1/\phi})$	$\dfrac{R_e(\psi)}{1+(i2\pi f\tau_0)^{\phi}}$	$\dfrac{R_{ct}(\psi)}{2\pi}\dfrac{\sin((1-\phi)\pi)}{\cosh\left(\phi\ln\left(\frac{\tau}{\tau_0}\right)\right)-\cos((1-\phi)\pi)}$
**WANG**	$\left(\left(R_i+\dfrac{1}{(j\omega C)^{\phi}}\right)^{-1}+\dfrac{1}{R_e}\right)^{-1}$	$\gamma(\ln\tau) = -\dfrac{1}{\pi}\left[Z''\left(\dfrac{1}{2\pi}e^{-\ln\tau+\frac{i\pi}{2}}\right)+Z''\left(\dfrac{1}{2\pi}e^{-\ln\tau-\frac{i\pi}{2}}\right)\right]$

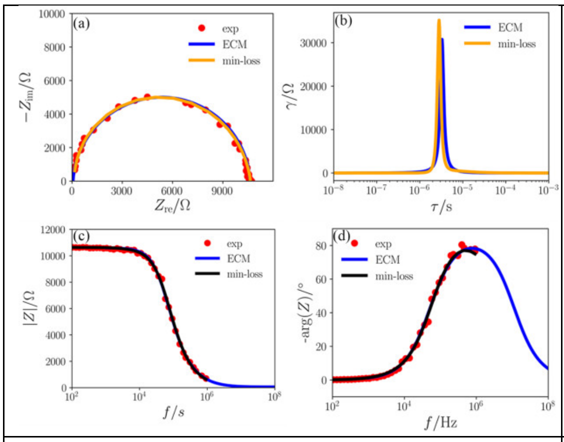

(A)

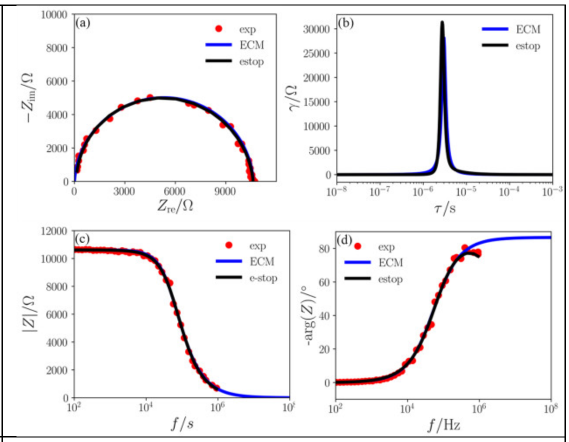

(B)

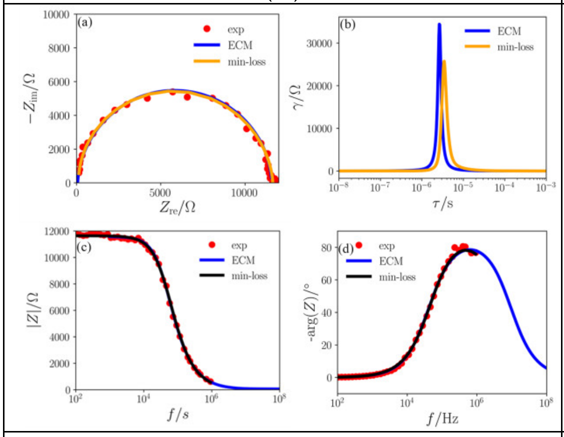

(C)

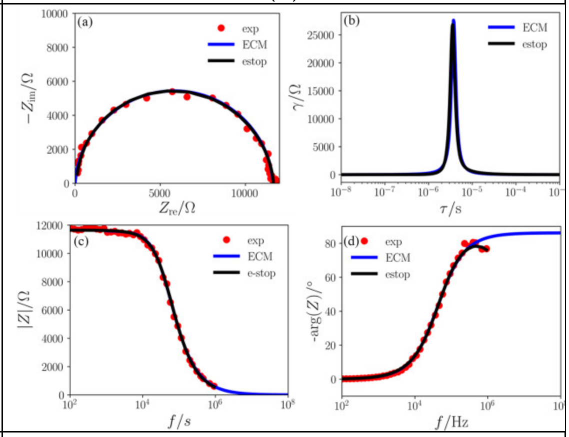

(D)

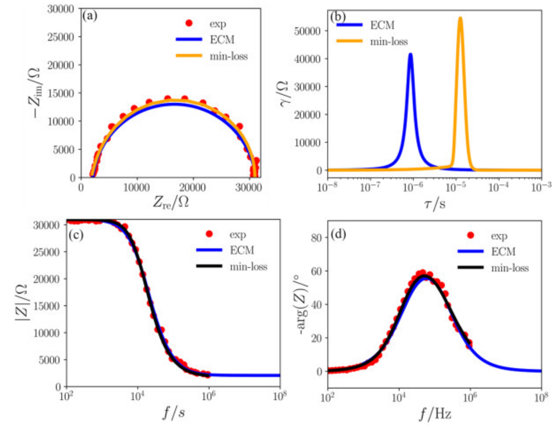

(E)

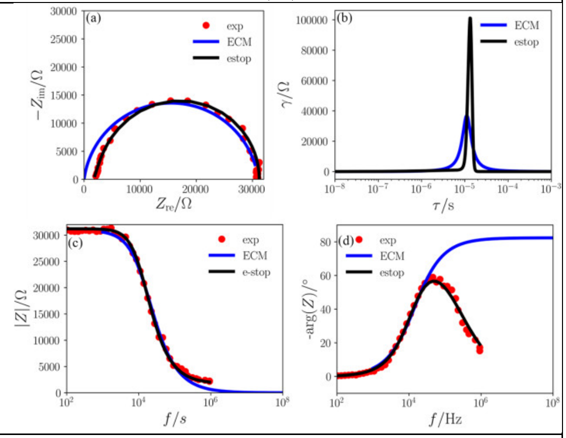

(F)

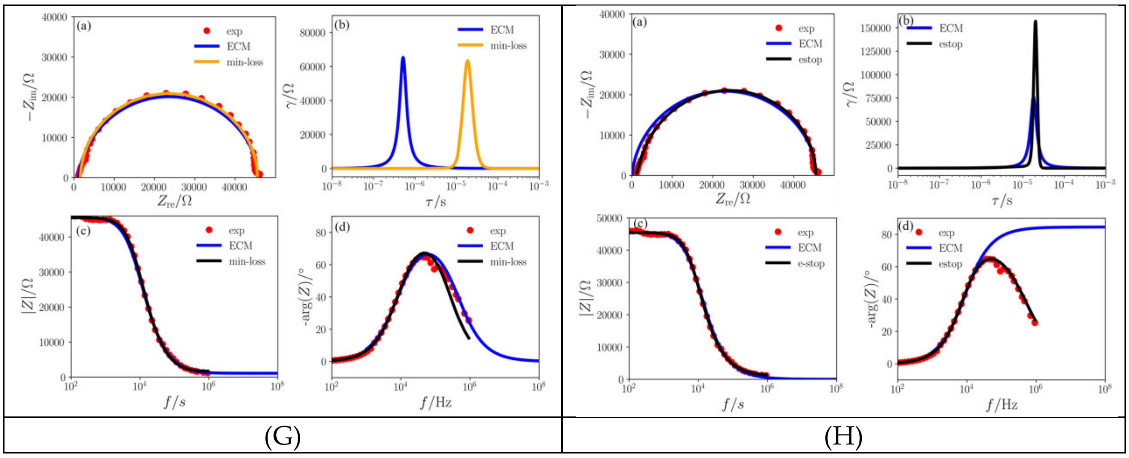

Figure S4. Nyquist fitting diagrams of the EIS data of LFP-AC-TOP—(A) Wang model, (B) ZARC model method, LFP-AC-Bottom—(C) Wang model, (D) ZARC model method, LFP-DC-TOP—(E) Wang model, (F) ZARC model method, and LFP-DC-Bottom—(G) Wang model, (H) ZARC model method.

Table S2. The electronic (σ_e) and ionic (σ_i) conductivity of the $LiFePO_4$ samples is calculated after fitting the curves.

**Samples**	MATLAB simplified Wang		Zview Wang model		DRT	
	σ_e(S/cm)	σ_i(S/cm)	σ_e(S/cm)	σ_i(S/cm)	σ_e(S/cm)	σ_i(S/cm)
SPS-AC Top	2.53×10^{-5}		2.53×10^{-5}	4.46×10^{-3}	2.53×10^{-5}	2.11×10^{-3}
SPS-AC Bottom	2.56×10^{-5}		2.57×10^{-5}	4.69×10^{-3}	2.56×10^{-5}	2.72×10^{-3}
SPS-DC Top	1.02×10^{-5}		1.01×10^{-5}	1.41×10^{-4}	1.02×10^{-5}	1.64×10^{-4}
SPS-DC Bottom	6.86×10^{-6}		6.85×10^{-6}	2.76×10^{-4}	6.93×10^{-6}	1.85×10^{-4}

References

1. Gao, S.; Su, Y.; Bao, L.; Li, N.; Chen, L.; Zheng, Y.; Tian, J.; Li, J.; Chen, S.; Wu, F. High-Performance $LiFePO_4$/C Electrode with Polytetrafluoroethylene as an Aqueous-Based Binder. *J. Power Sources.* **2015**, *298*, 292–298. https://doi.org/10.1016/j.jpowsour.2015.08.074.
2. Zhu, W.; Wang, Y.; Liu, D.; Gariépy, V.; Gagnon, C.; Vijh, A.; Trudeau, M. L.; Zaghib, K. Application of Operando X-Ray Diffractometry in Various Aspects of the Investigations of Lithium/Sodium-Ion Batteries. *Energies.* **2018**, *11* (11), 2963. https://doi.org/10.3390/en11112963.
3. Rosaiah, P.; Hussain, O. M. Microscopic and Spectroscopic Properties of Hydrothermally Synthesized Nano-Crystalline LiFePO4 Cathode Material. *J. Alloys Compd.* **2014**, *614*, 13–19. https://doi.org/10.1016/j.jallcom.2014.06.072.
4. Burba, C. M.; Frech, R. Raman and FTIR Spectroscopic Study of Li_xFePO_4 ($0 \leq x \leq 1$). *J. Electrochem. Soc.* **2004**, *151* (7), A1032–A1038. https://doi.org/10.1149/1.1756885.
5. Wang, C.; Hong, J. Ionic/Electronic Conducting Characteristics of $LiFePO_4$ Cathode Materials. *Electrochem. Solid-State Lett.* **2007**, *10* (3), 65–69. https://doi.org/10.1149/1.2409768.
